# Meta-analysis of trigger timing in normal responders undergoing GnRH antagonist ovarian hyperstimulation protocol

**DOI:** 10.1186/s13048-024-01379-3

**Published:** 2024-03-05

**Authors:** Qijun Xie, Danyu Ni, Sisi Chen, Wenjie Zhang, Jue Wang, Xiufeng Ling, Rong Shen

**Affiliations:** 1 Department of Reproductive Medicine, Nanjing Women and Children’s Healthcare Hospital, Women’s Hospital of Nanjing Medical University, No. 123 Tianfei Lane. Mochou Road, Qinhuai District, Nanjing, 210004 Jiangsu China; 2 Medical Affairs and Outcomes Research, Organon Research and Development, Organon (Shanghai) Pharmaceutical Technology Co., Ltd., Shanghai, 20003 China; 3 Systematic Review Solutions Ltd, Tianjin, 300000 China

**Keywords:** Ovarian hyperstimulation, GnRH antagonist, Trigger timing, Final oocyte maturation, Meta-analysis

## Abstract

**Importance:**

The first meta-analysis focused only on gonadotropin-releasing hormone (GnRH) antagonists, which helped determine the effect of delay trigger on pregnancy outcomes.

**Objective:**

To evaluate the impact of delay trigger compared with standard trigger in normal responders undergoing GnRH antagonist protocol in improving pregnancy outcomes.

**Methods:**

Studies published before April 2023 in PubMed, EMBASE, Cochrane Library, Web of Science, CNKI, Wanfang, VIP and CBM databases were searched. Randomized controlled trials (RCTs) and cohort studies conducted in normal responders reporting the efficacy of delay trigger using GnRH antagonist protocol were included. Data were combined to calculate mean differences (MD) for continuous variables and odd ratios (OR) for categorical variables with their corresponding 95% confidence intervals (CIs). Heterogeneity was assessed using Cochran’s Q test.

**Results:**

Endpoints, including clinical pregnancy rate (CPR), live birth rate (LBR), the number of oocyte retrievals and embryos, and fertilization rate, were analyzed. Six (6) clinical studies (4 RCTs and 2 cohort studies) with 1,360 subjects were included. The pooled results showed that the number of oocyte retrievals (MD: 1.20, 95% CI: 1.10, 1.30, *p* < 0.01), fertilization rate (MD: 0.64, 95% CI: 0.29, 0.99, *p* < 0.01) and days of stimulation (MD: 0.95; 95% CI: 0.54, 1.37; *p* < 0.01) in the delay trigger group was significantly higher than that in the standard trigger group. However, there was no significant difference in the number of embryos (MD: 0.19, 95% CI: -0.29, 0.67, *p* = 0.44), CPR (OR: 1.12; 95% CI: 0.72, 1.75; *p* = 0.062), and LBR (OR: 1.23; 95% CI: 0.90, 1.66; *p* = 0.19) between the two trigger groups.

**Conclusion:**

Delaying trigger time in GnRH antagonist protocol increased the number of oocytes retrieved but not the number of embryos. Furthermore, delay trigger shot was not associated with a clinical benefit towards CPR and LBR in women who underwent fresh embryo transfer cycles.

**Trial registration:**

The International Prospective Register of Systematic Reviews (PROSPERO), registration number: CRD42023413217.

**Supplementary Information:**

The online version contains supplementary material available at 10.1186/s13048-024-01379-3.

## Introduction

During controlled ovarian hyperstimulation (COH), supraphysiological gonadotropins (Gn) administration facilitated multiple follicle development, which may lead to early endogenous pituitary surge before a majority of follicles become mature in approximately one-third of patients [1, 2]. Therefore, a gonadotropin-releasing hormone (GnRH) analogue was administrated to inhibit premature luteinizing hormone (LH) surge and premature ovulation during COH. GnRH antagonists could reversibly inhibit endogenous LH without pituitary down-regulation, creating an unequal follicular dynamic and reproductive endocrinology condition compared to GnRH agonist protocol [3]. Due to the insufficient endogenous LH after GnRH antagonist injection, hCG was administrated to trigger final oocyte maturation and corpus luteal formulation.

Besides the trigger drug, the timing of triggering is critical for reproductive outcomes in GnRH antagonist protocol. The decision regarding the trigger timing on follicle size involves several factors, including the number of developing follicles in the cohort, hormone levels on the day of pursued trigger, the duration of stimulation, the patient’s clinical and economic burden, the experience with previous cycles, and the IVF center practice pattern [4]. For a GnRH antagonist protocol, the trigger drug is usually administrated when ≥ 3 follicles reach a diameter ≥ 17 mm or when ≥ 2 follicles reach 18 mm in diameter [5]. However, this criterion was considered standard trigger timing only for normal responders but did not apply to high and low responders due to differences in developing follicle cohort.

During clinical practice, many IVF practitioners may wait one to two days after the patient meets the standard criteria to get more mature oocytes or just for convenience to avoid weekend procedures. However, there is a conflicting opinion on delay trigger. Even if the delay may produce more oocytes [6, 7], it also comes with additional risks, including early ovulation and premature progesterone elevation. The rise in progesterone on the trigger day is associated with a lower live birth rate (LBR) in the fresh embryo transfer (ET) cycle due to impaired endometrial receptivity [8]. Therefore, determining the optimal trigger timing in GnRH antagonists is critical, especially in the fresh ET cycle.

Previously, a meta-analysis [9] compared the standard trigger timing with 1- or 2-day late trigger. Study results suggested that prolonging the follicular phase by delaying hCG administration increased oocyte retrieval number but did not increase LBR. However, this meta-analysis included GnRH agonist and antagonist protocols without extensive pooled analyses on GnRH antagonists. As the follicular dynamic and reproductive endocrine changes differ between the two protocols, the embryonic and pregnancy outcomes based on the trigger timing in the GnRH-antagonist protocol merit clinical investigation. Several studies have been conducted to seek more evidence. Daver et al. and Awonuga et al. indicated that delay triggers were not associated with a higher number of oocytes and an increased clinical pregnancy rate (CPR) [6, 7]. Unfortunately, the sample size in the two studies was small and not statistically powered to give a robust conclusion. Therefore, summarizing the evidence in the timing of the trigger shot in the GnRH antagonist protocol is important.

This meta-analysis aimed to investigate the effect of delay trigger compared with standard trigger for normal responders undergoing GnRH antagonist protocol in improving pregnancy outcomes. As the delay duration varied, which might have influenced the outcome, we used subgroup analysis with 24-hour and 48-hour delays to exclude the interference.

## Methods

### Eligibility criteria

Published clinical studies were required to meet the following criteria to be eligible for the meta-analysis: (1) subjects were infertility women undergoing IVF/ICSI with GnRH antagonist protocol; (2) studies reported the efficacy of delay trigger timing (24 or 48 hour delay) and standard trigger timing; (3) efficacy endpoint was evaluated by analysis of the number of oocytes retrieved, number of embryos, CPR and LBR; and (4) the study was a randomized controlled trial (RCT) or a cohort study. Search results were restricted to articles written in English and Chinese, and no limitations regarding the publication date were applied.

Articles were excluded if: (1) studies were conducted in specific populations, including oocyte donors, high responders, poor responders, and advanced-age women (> 35 years old); (2) studies investigated trigger timing but subjects were grouped based on other criteria ( e.g. leading follicle diameter, ratio of dominant follicles) instead of trigger timing (standard vs. delay) ; (3) studies repeated in different databases; (4) studies did not present essential or clear information, included specification of trigger timing in each group and pregnancy outcomes such as CPR and LBR; and (5) study results were from unpublished manuscripts and conference abstracts. If multiple published reports from the same study were detected, only the publication with the most detailed information for original data and outcomes was included.

### Search strategies

A systematic literature search was conducted according to the Preferred Reporting Items for Systematic Reviews and Meta-Analysis (PRISMA) guidelines [10]. PubMed, EMBASE, Cochrane Library, Web of Science, CNKI, Wanfang, VIP and CBM databases were comprehensively and systematically searched for potentially eligible studies. The first search was conducted up to May 2022 and later supplemented from May 2022 to April 2023. The search strategy used the following main search terms: ovarian hyperstimulation, criteria for triggering, trigger timing, time of hCG, oocyte triggering, time of oocyte maturation, and follicle size. The detailed search strategy for each database is presented in Additional File 1. We did not add ‘GnRH antagonist’ in the search strategy to avoid missing studies because many studies did not report COH protocol in the abstract. Furthermore, clinical trial registration websites, references of the selected studies, or relevant review articles were reviewed to find as many relevant studies as possible.

### Literature screening

We used Endnote for de-duplication and literature screening. Based on the inclusion and exclusion criteria, two reviewers (Wenjie Zhang and Sisi Chen) independently screened titles, abstracts, and full texts of studies identified during searches. Differences between reviewers over the title and abstract screening, full-text review, and reasons for exclusion were reconciled with a third reviewer (Qijun Xie).

### Data extraction and quality assessment

Data extraction and risk of bias assessment were performed independently by two members (Wenjie Zhang and Sisi Chen), and a third expert (Qijun Xie) resolved the disagreement, if any. Data extraction tables were constructed and agreed upon between the authors. The selected studies were comprehensively examined and grouped according to the topic of interest, and relevant data were entered into the tables. The necessary information extracted from the available literature, including first author’s name, publication year, study sites, study period, methodology, patients’ characteristics, grouping criteria in the intervention and control group, sample size, fertilization method, trigger drug and dose, embryo transfer strategy, outcome measures, and summary of findings. The primary outcomes evaluated were CPR and LBR. Secondary endpoints covered oocytes retrieved and the number of embryos. In addition, we have focused on fertilization rate, estradiol level, progesterone level, Gn duration and total Gn dosage. We synthesized the outcomes for delay by 1 day and 2 days.

For RCTs, the risk of bias was further assessed with the Cochrane Risk of Bias assessment tool [11], including the following seven domains: generation of a randomization sequence, allocation concealment, blinding, incomplete outcome data, selective reporting, and other biases. Moreover, for cohort studies, we use the Newcastle-Ottawa Scale (NOS), which ranges from 0 to 9 stars and judges each study regarding three aspects: selection, comparability, and outcomes of interest, with higher stars indicating a lower risk of bias.

### Statistical analysis

Data were analyzed using the Cochrane Review Manager (RevMan) 5.3 to analyze the extracted data for summary effect estimates and generate forest plots. Individual and pooled statistics were expressed as mean differences (MD) and 95% confidence intervals (CI) for continuous variables and odd ratios (OR) for categorical variables with their corresponding 95% CIs. Although the fertilization outcome was expressed as a rate overall, this outcome was assessed as a continuous variable for the individual subject. Therefore, pooled statistics were expressed as MD and 95% CIs. Statistical heterogeneity was assessed using Cochran’s Q test. If there was no substantial statistical heterogeneity (I^2^ > 50%) [12], data were combined using the fixed-effect model; otherwise, the heterogeneity was evaluated using the random-effect model. The causes of heterogeneity were analyzed and processed using subgroup analysis. We pooled trials by standard trigger versus 24-hour trigger group and standard trigger versus 48-hour trigger group. In addition, we analyzed subgroups of the study design (RCTs and cohort studies) (Additional File 2). Moreover, we also analyzed including RCTs at low risk of bias (Additional File 3). The cut-off for statistical significance was set at a two-sided *p* < 0.05. This study is registered with PROSPERO, CRD42023413217 (https://www.crd.york.ac.uk/PROSPERO/display_record.php?RecordID=413217).

## Results

### Literature screening

**Fig. 1:**
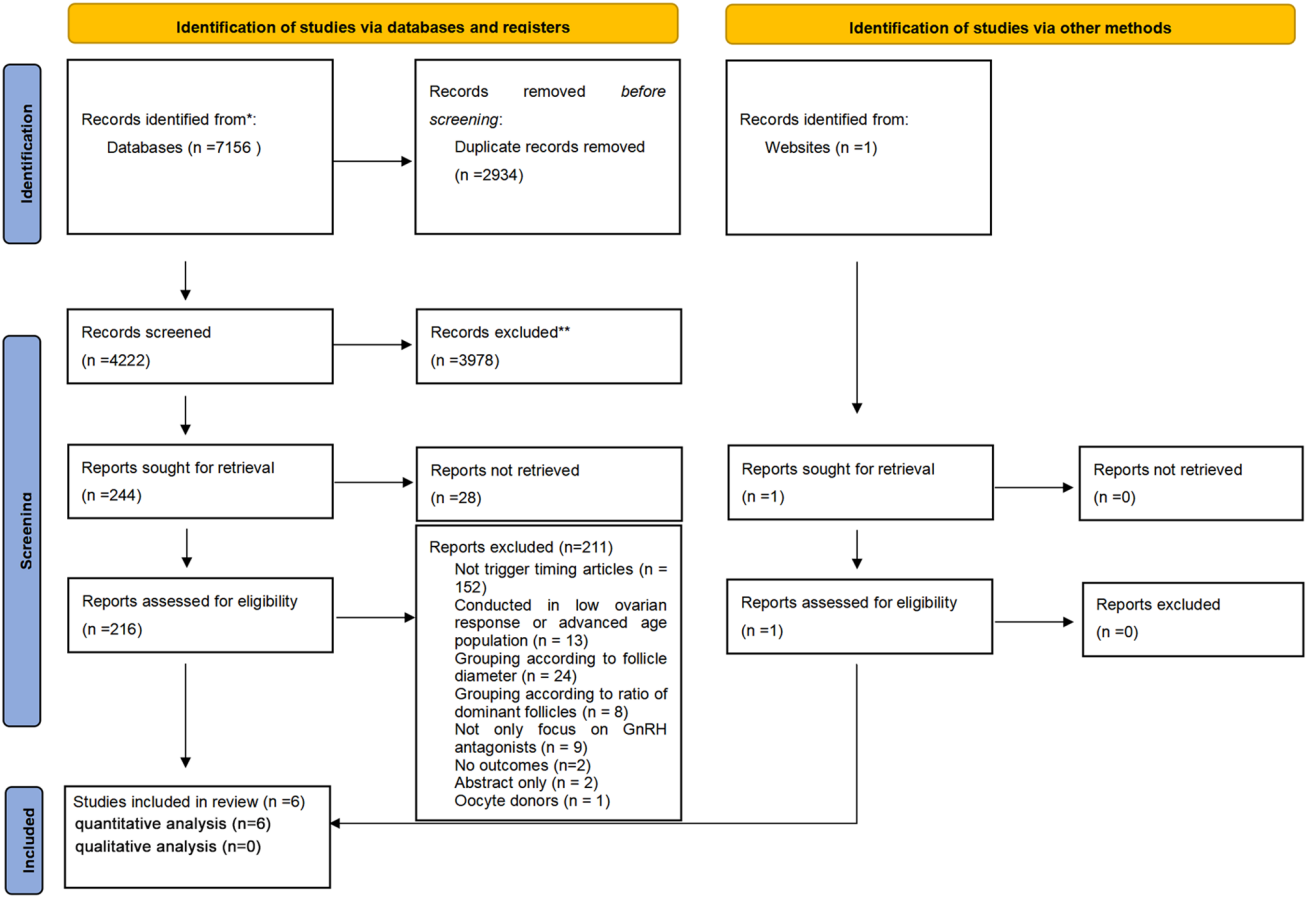
PRISMA flow diagram

The initial search identified 7156 potentially relevant manuscripts and 1 additional article [13]. Among them, 2934 duplicates were removed, 3978 manuscripts were excluded after reviewing the titles and abstracts, and 28 reports were not retrievable. A full-text review was performed for the remaining 216 articles, in which 211 were discarded for non-conformity with the prespecified inclusion and exclusion criteria after full-text review. Finally, 6 articles (including 1 additional article from the website) were considered eligible for the meta-analysis, which were all quantitative analyses without qualitative analysis. The Preferred Reporting Items for Systematic Reviews and Meta-Analysis (PRISMA) [14] flowchart outlining the study selection procedure is shown in Fig. 1.

### Characteristics of included studies

These studies [6, 7, 13, 15–17] were conducted between 2002 and 2016 and included 1,360 participants. Four (4) [6, 15–17] were RCTs, and 2 were [7, 13] retrospective studies. Three (3) studies defined the standard trigger timing as two or more follicles ≥ 17 mm in diameter [13, 15, 17], two defined as two or more follicles ≥ 18 mm [6, 7], one study defined as three or more follicles ≥ 16 mm in diameter [16]. Four (4) studies compared the standard trigger with the 24-hour delay trigger, 2 studies compared the standard trigger with the 48-hour delay trigger. All 6 trials applied both ICSI and IVF treatment cycles, and hCG was administrated for trigger and followed by fresh ET in all studies. There were 670 subjects in the standard trigger group and 690 in the delay trigger group. The details of 6 trials are described in Table 1.

**Table 1:** Characteristics of included studies

First author, publication year	Methodology	Single center or multi center	Location	Study period	Definition of standard trigger timing	Intervention and Comparison	Sample Size	Trigger drug and dose	Fertilization method	Embryo transfer strategy	Primary outcome	Findings
Kolibianakis, 2004 [15]	RCT	single center	Belgium	2002.5-2003.4	at least three follicles were 17 mm	standard trigger VS 48 h-delay trigger	194/196	hCG,10000 IU	IVF/ICSI	fresh embryo	ongoing pregnancy rate	Prolongation of the follicular phase in patients stimulated with rec-FSH and GnRH antagonists for IVF does not affect oocyte or embryo quality but is associated with a significantly lower ongoing pregnancy rate.
Tremellen1, 2010 [13]	Cohort	single center	Australia (Adelaide)	2008	two or more follicles ≥ 17 mm in diameter, with the majority of follicles being ≥ 14 mm	standard trigger VS 24 h-delay trigger	221/251	hCG,5000 IU	IVF/ICSI	fresh embryo	live birth rate	It is possible to safely avoid weekend oocyte retrievals (ORs) during GnRH antagonist cycles by simply advancing an ideal Saturday OR to Friday, and delaying an ideal Sunday OR to Monday, without adversely impacting on IVF live birth outcomes.
Kyrou, 2011 [16]	RCT	single center	Belgium	2010.1-2011.4	three or more follicles of size ≥ 16 mm	standard trigger VS 24 h-delay trigger	52/54	hCG,10000 IU	IVF/ICSI	fresh embryo	ongoing pregnancy rate.	Earlier administration of hCG is not associated with the probability of pregnancy in cycles stimulated with recombinant FSH and GnRH antagonists.
Morley, 2012 [17]	RCT	single center	United Kingdom	2002–2007	≥ 3 follicles ≥ 17 mm in diameter	standard trigger VS 24 h-delay trigger VS 48 h-delay trigger	39/37/31	hCG,10000 IU	IVF/ICSI	fresh embryo	the number of oocytes retrieved	Delaying hCG administration had no significant negative impact upon morphological quality of embryos, availability of surplus embryos for freezing or pregnancy outcomes. Postponing hCG may enable increased flexibility of cycle scheduling to avoid weekend procedures.
Davar, 2017 [6]	RCT	single center	Iran	2016.8-2016.10	at least 3 follicles with ≥ 18 mm diameters	standard trigger VS 24 h-delay trigger	43/42	——	IVF/ICSI	fresh embryo	Number of metaphase II oocytes, number of fertilized oocytes, number of embryo formation.	Delaying of triggering oocyte maturation by 24 h in antagonist protocol with not-elevated progesterone (progesterone ≤ 1 ng/ml) have not beneficial nor harmful effect on the number of mature oocytes (MII) and other in vitro fertilization cycle characteristics.
Awonuga, 2018 [7]	Cohort	single center	USA	2003.1-2009.12	two mature follicles, defined as ≥ 18 mm	standard trigger VS delay trigger	121/79	——	IVF/ICSI	fresh embryo	-	delaying hCG administration to allow further growth of the medium-sized follicles added further days of superovulation and cost without improvement in CPR and LBR.

### The methodological quality of the included studies

**Fig. 2:**
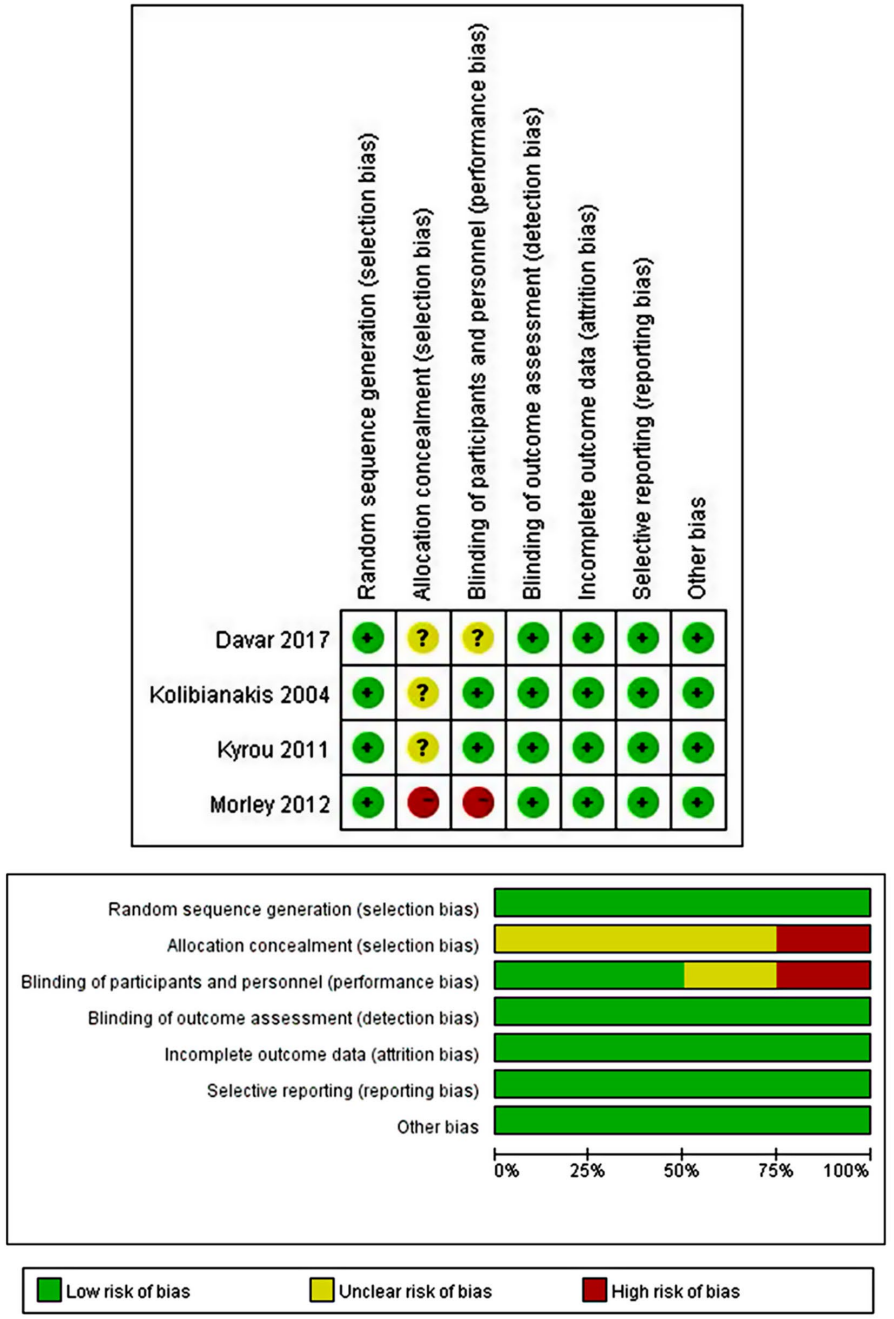
The risk bias evaluation of RCTs included in the meta-analysis. The included RCTs had a low risk of bias for random sequence generation, blinding or participants and personnel, blinding of outcome assessment, incomplete outcome data, selective reporting, and other biases (green bars for corresponding items). For allocation concealment, 3 RCTs did not report how allocation was concealed, and one did not conceal. Therefore, 25% of included RCTs had a high risk of bias, and 75% had an unclear risk of bias (a yellow and red bar). For blinding of participants and personnel, 1 RCT did not report if blinding was conducted, and 1 was open-label. Therefore, 25% of included RCTs had a high risk of bias, 25% had an unclear risk of bias, and 50% had a low risk of bias (a green, yellow, and red bar)

Among the included 4 RCTs, all had a randomized allocation. Three (3) trials did not provide details for allocation concealment [6, 15, 16]. In addition, 1 RCT was an open-label study and clearly described that the clinicians and patients were not blinded to the allocated treatment arm [17]. Only 1 RCT described double-blinding to their personnel and participants [15] (see Fig. 2). Among the two cohort studies, one scored 8 [7], and the other scored 7 [13].

### Meta-analysis

#### Oocytes retrieved

**Fig. 3:**
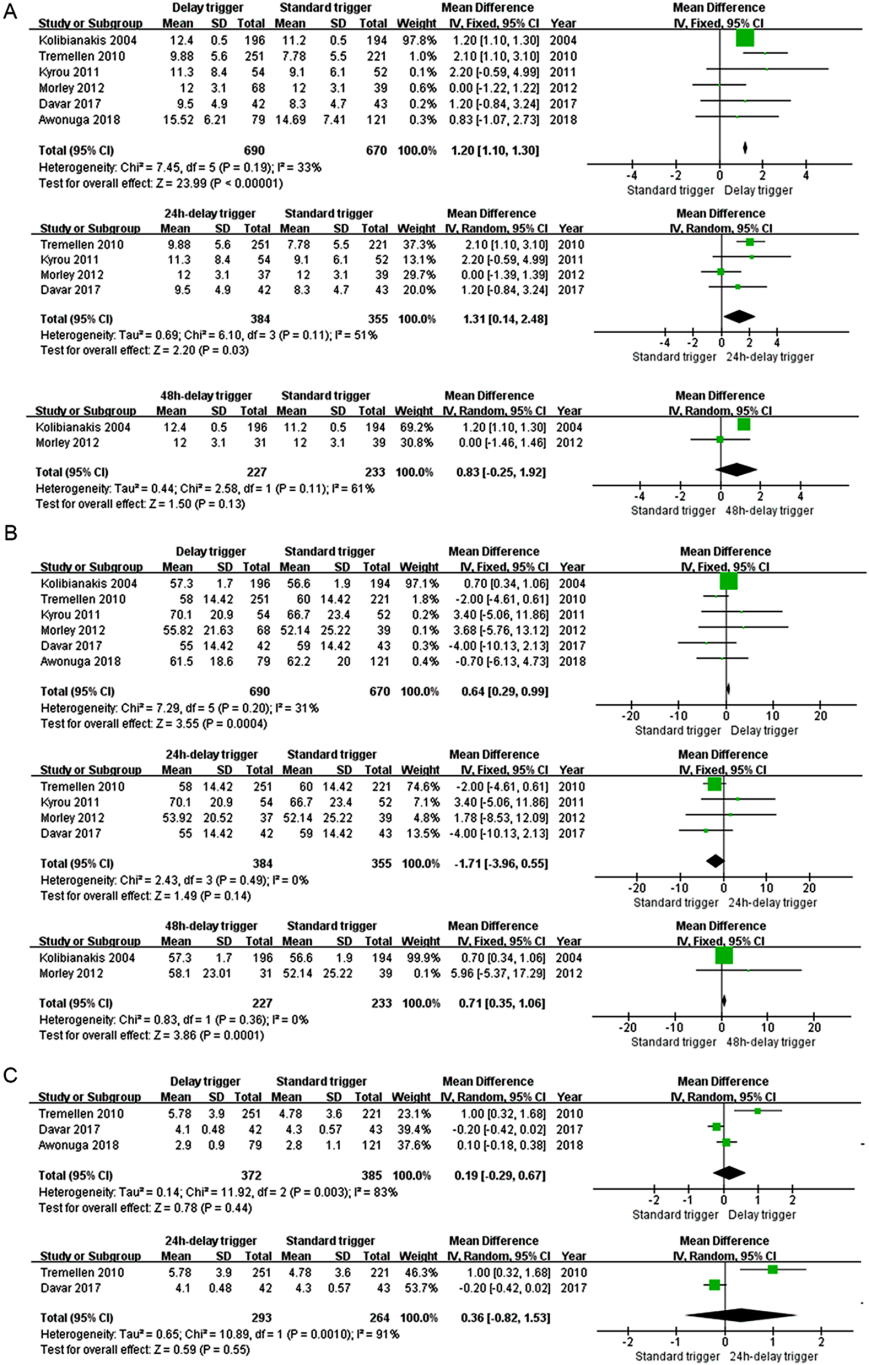
Comparison of the number of oocytes retrieved (**A**), the fertilization rate (**B**) and the number of embryos (**C**) for the standard and delay trigger groups

Six (6) studies [6, 7, 13, 15–17] and 1,360 subjects were included. The number of oocyte retrievals in the delay trigger group was significantly higher than that in the standard trigger group (MD: 1.20, 95% CI: 1.10, 1.30, *p* < 0.01), with no statistical heterogeneity (*p* = 0.19, I^2^ = 33%; Fig. 3A). Moreover, the subgroup analysis found similar results between the 24-hour delay and the standard trigger groups (MD: 1.31, 95% CI: 0.14, 2.48, *p* = 0.03). Nevertheless, there was no significant difference between the 48-hour delay and the standard trigger groups (*p* = 0.13).

### Fertilization rate

Six (6) [6, 7, 13, 15–17] eligible studies and 1,360 subjects were included. The fertilization rate was higher in the delay trigger group compared with the standard trigger group (MD: 0.64, 95% CI: 0.29, 0.99, *p* < 0.01) and in the 48-hour delay trigger group compared with the standard trigger group (MD: 0.71, 95% CI: 0.35, 1.06, *p* < 0.01), while there was no significant difference between the standard trigger and the 24-hour delay trigger groups (*p* = 0.14). The statistically significant heterogeneity was not observed in total (*p* = 0.20, I^2^ = 31%) and in the subgroup of the 24-hour delay (*p* = 0.14, I^2^ = 0%) and the 48-hour delay trigger groups (*p* = 0.36, I^2^ = 0%) (see Fig. 3B).

### Number of embryos

Three (3) trials [6, 7, 13] and 757 subjects were included. There was no significant difference between the delay and the standard trigger groups (MD: 0.19, 95% CI: -0.29, 0.67, *p* = 0.44) with significant heterogeneity (*p* = 0.003, I^2^ = 83%). Furthermore, in the subgroup analysis, results were similar between the 24-hour delay and the standard trigger groups (MD: 0.36, 95% CI: -0.82, 1.53, *p* = 0.55) when a random-effect model was used (*p* = 0.001, I^2^ = 91%). See Fig. 3C.

### Clinical pregnancy rate

**Fig. 4:**
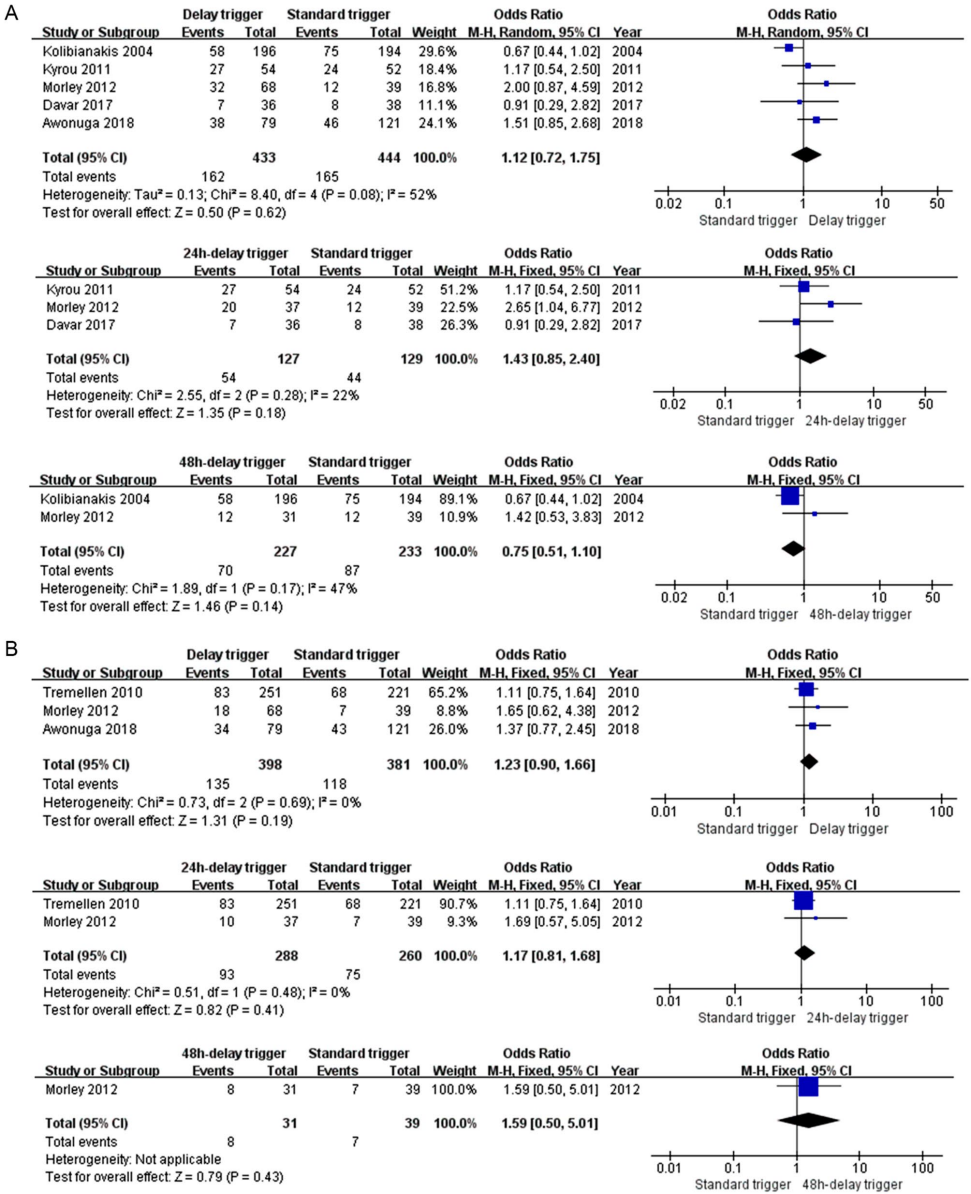
Comparison of the clinical pregnancy rate (**A**) and the live birth rate (**B**) for the standard and delay trigger groups

Five (5) [6, 7, 15–17] eligible studies and 888 subjects were included. There was no significant difference between the delay and standard trigger groups (OR: 1.12, 95% CI: 0.72, 1.75, *p* = 0.062). Furthermore, in the subgroup analysis, similar results were shown in the 24-hour delay trigger group versus the standard trigger group (MD: 1.43, 95% CI: 0.85, 2.40, *p* = 0.18) and in the 48-hour delay trigger group versus the standard trigger group (MD: 0.75, 95% CI: 0.51, 1.10, *p* = 0.14). No statistical heterogeneity was observed in the subgroups of the 24-hour delay trigger group (*p* = 0.28, I^2^ = 22%) and the 48-hour delay trigger group (*p* = 0.17, I^2^ = 47%) (see Fig. 4A).

### Live birth rate

Three (3) trials [7, 13, 17] and 779 subjects were included. There was no statistical difference in the delay trigger group versus the standard trigger group (MD: 1.23, 95% CI: 0.90, 1.66, *p* = 0.19) or the 24-hour delay trigger group versus the standard trigger group (MD: 1.17, 95% CI: 0.81, 1.68, *p* = 0.41). Heterogeneity in both groups was 0% (*p* = 0.69 and *p* = 0.48, respectively). Only one study compared the 48-hour delay and the standard trigger groups (MD: 1.59, 95% CI: 0.50, 5.01, *p* = 0.43) (see Fig. 4B).

### Others: estradiol level and progesterone level on trigger day, Gn duration, and total Gn dosage

We also concluded meta-analyses for estradiol level, progesterone level, Gn duration, and total Gn dosage. The 48-hour delay trigger group have a higher estradiol level (MD: 376.00, 95% CI: 361.11, 390.89, *p* < 0.01) and higher progesterone level (MD: 0.40, 95% CI: 0.38, 0.42, *p* < 0.01) than the standard trigger group. The delay trigger group, whether it was 24 or 48 h of delay, had a longer Gn duration but no significant difference compared with the standard trigger group. Nevertheless, in the subgroup analysis, the 24-hour delay trigger group (MD: 143.00, 95% CI: 12.85, 273.14, *p* = 0.03) and 48-hour delay trigger group (MD: 324.31, 95% CI: 30.77, 617.86, *p* = 0.03) have more dosage of total Gn than the standard trigger group. Details are shown in Additional File 4.

## Discussion

This meta-analysis showed no statistically significant difference in CPR and LBR between delay and standard trigger timing in women with a GnRH antagonist for ovarian hyperstimulation. Meanwhile, our results suggested that although the number of oocyte retrievals, but not the embryos, in the delay trigger group, was significantly higher than that in the standard trigger group. The results of the pooled RCTs and low-risk RCTs, respectively, were consistent with those of all included studies.

Regarding the likelihood of achieving high clinical efficacy, our first concern was whether the delay trigger could retrieve more oocytes. The results from the present meta-analysis indicated that there were possibly more oocytes after delay trigger in the GnRH antagonist protocol (Fig. 3A). This finding is reasonable, as delay trigger administration to prolong the follicle phase permits the continuous growth of the follicle cohort during this period, resulting in more oocytes at retrieving. Of note, in the subgroup analysis, we found that the 24-hour delay trigger obtained more oocytes [6, 13, 16, 17], but this delay trigger became invalid when prolonged to 48 h [15, 17]. It must be cautious to interpret this negative result in the 48-hour subgroup analysis because only two studies were included, and their results were inconsistent. The non-significant finding in the 48-hour subgroup analysis is from Morley’s study [17]. Even though the median oocytes on the collection day under ultrasound were 17 and 14 in the 48-hour delay and the standard trigger groups, the median retrieved oocytes were 12 in both groups. Considering this result, a hypothesis is that there may be a threshold requirement and cut-off point in the GnRH antagonist protocol, which the length of delay will impact the number of oocytes. A hypothetical cause of these results is that an excessively prolonged follicular phase might increase the early ovulation, leading to cycle cancellation in some patients, then compromising the advantage of mean retrieved oocytes [7, 15]. Furthermore, the overdevelopment of some follicles at a 48-hour delay might lead to follicular atresia, which may reduce oocyte retrieval [18, 19]. Future studies are still in need to support this hypothesis.

Our second concern was whether the delay trigger could retrieve more embryos. The present meta-analysis showed that despite the increase in oocyte number after the delay trigger, the number of embryos did not increase accordingly. The results in the subgroup analysis were consistent. An explanation is that prolonging folliculogenesis could be detrimental to oocyte quality and consequently reduce the formation of transferable embryos [20]. For example, an in vitro study in cattle found a more significant proportion of atretic follicles in heifers with an extended follicular phase [19].

Besides the number of oocytes and embryos, the primary objective was to investigate whether a delay trigger shot could optimize the pregnancy outcome. The results from the present meta-analysis put in serious doubt the delay trigger in improving pregnancy outcomes as there were no significant differences in LBR or CPR. The pooled result is consistent with most studies included in the analysis. However, three studies only reported results from the 24-hour delay trigger [6, 16, 17], and two [6, 15] investigated the groups with the 48-hour delay trigger. Of note, in the 48-hour subgroup analysis, although there was no statistical difference in CPR (OR: 0.75, 95% CI: 0.51, 1.10, *p* = 0.14), there might be a trend to prefer the standard trigger rather than the 48-hour delay. We did not perform a pooled analysis of ongoing pregnancy rate (OPR) as only two studies included OPR as an outcome for analysis. Nevertheless, Efstratios and associates also found that the 48-hour delay group had a significantly lower OPR [15]. One possible reason for this result was that an unduly delay trigger could impact endometrial receptibility and then influence embryo implantation. High levels of estradiol production occur earlier in the COH cycle than in natural cycles, which induces progesterone receptors in the endometrium during the follicular phase and, thus, advances endometrial development [21]. In addition, maturated follicles could produce excessive amounts of progesterone [22], which might further advance endometrial development. This hypothesis was supported by the 48-hour subgroup analysis result, which showed that the estradiol and progesterone levels significantly increased in the 48-hour delay trigger group (Additional File 4). Based on the above hypothesis, it seems interpretable why the 48-hour delay trigger ended up with a lower pregnancy rate in Efstratios M’s study.

Cost remains one of the most significant barriers to accessing and using infertility services [23]. Pregnancy outcomes were similar in both groups in our study, but the delay trigger resulted in longer stimulation durations and a higher total dose of Gn drug use. In addition, the delay trigger resulted in higher numbers of oocytes, which is associate with longer procedure duration and time spent in the post anaesthesia care unit. [24]. Therefore, delaying the trigger timing may increase the financial burden.

There are several limitations in our meta-analysis. Only six studies were included in our study, which seems insufficient to yield a powerful conclusion. Because two cohort studies were included, selection bias was unavoidable [7, 13]. The definition of standard trigger timing varied; three studies used the criteria of 2 or more follicles ≥ 17 mm [13, 15, 17]. One study used the criteria of 3 or more follicles ≥ 16 mm [16]. Two studies used criteria of 2 or more follicles ≥ 18 mm [6, 7]. This variation may lead to measurement bias. Furthermore, two studies did not directly report mean or standard deviation (SD) for the continuous variables [7, 17]. We converted the median (and range) to mean and SD or estimated the SD, making it difficult to pool data.

## Conclusion

In summary, delaying the trigger time in the GnRH antagonist protocol improved the number of oocytes retrieved but not the number of embryos. Further, delaying the trigger did not clinically benefit clinical pregnancy and live birth rates with increased total Gn dose in women with fresh embryo transfer cycle. Due to the limited number of included studies, well-designed randomized controlled trials with a large sample size must further confirm these findings.

### Electronic supplementary material

Below is the link to the electronic supplementary material.

Supplementary Material 1

Supplementary Material 2

Supplementary Material 3

Supplementary Material 4

## Data Availability

The datasets supporting the conclusions of this article are included within the article and its additional files.

## Abbreviations

CIs: Confidence intervals
COH: Controlled ovarian hyperstimulation
CPR: Clinical pregnancy rate
ET: Embryo transfer
GnRH: Gonadotropin-releasing hormone
LBR: Live birth rate
LH: Luteinizing hormone
MD: Mean differences
OR: Odd ratios
PACU: Post-anesthesia care unit
PRISMA: Preferred Reporting Items for Systematic Reviews and Meta-Analysis
RCTs: Randomized controlled trials
