# Supplementary material for: Meta-analysis of trigger timing in normal responders undergoing GnRH antagonist ovarian hyperstimulation protocol
Source: J Ovarian Res. 2024 Mar 5;17:56. doi: 10.1186/s13048-024-01379-3 (PMC10913352; doi:10.1186/s13048-024-01379-3)
Supplement: Supplementary file 1 — Supplementary Material 1 [file 13048_2024_1379_MOESM1_ESM.docx]

**Additional file 1.** Search strategy for each database.

**Pubmed**

1. "Reproductive Techniques, Assisted"[Mesh] OR ART[tw] OR artificial inseminat*[tw] OR intrauterine insemination[tw] OR fertility preservat*[tw] OR embryo disposit*[tw] OR Embryo Transfer*[tw] OR in-vitro fertilization*[tw] OR fertilization in-vitro[tw] OR intracytoplasmic sperm injection*[tw] OR ovulation induction*[tw] OR "oviduct* insemination"[tw] OR "IUI"[tw] OR "intra uterine insemination"[tw] OR Gamete Intrafallopian Transfer[tw] OR In-Vitro Oocyte Maturation*[tw] OR Oocyte Donation*[tw] OR Oocyte Retrieval*[tw] OR Ovulation Induction*[tw] OR Superovulation*[tw] OR Zygote Intrafallopian Transfer*[tw] OR Assisted reproduction*[tw] OR embryo transplant*[tw] OR frozen embryo*[tw] OR intracytoplasmic sperm injection*[tw] OR single sperm injection*[tw] OR pregnancy rate[tw] OR implantation rate[tw] OR live birth rate[tw] OR pregnancy outcome[tw] OR ovarian reserve[tw] OR ovulation induction*[tw] OR ovarian stimulation*[tw] OR IVF[tw] OR ICSI[tw] OR "ovarian hyperstimulation*"[tw] OR Ovarianstimulation*[tw] OR induced ovulation*[tw] OR ovulation inducing[tw] OR COH[tw] OR hyperovulation*[tw]
2. ((hCG[tw] OR "Human Chorionic Gonadotrophin"[tw] OR "oocyte collection*"[tw] OR "oocyte maturation*"[tw] OR "oocyte retrieval"[tw]) AND (timing[tw] OR trigger*[tw])) OR hCG administration*[tw] OR Oocyte trigger*[tw] OR ovulation trigger*[tw] OR stimulation duration*[tw]
3. #1 and #2

**EMBASE**

1. 'infertility therapy'/exp OR (ART OR IVF OR ICSI OR COH OR IUI OR hyperovulation* OR ((artificial OR intrauterine* OR oviduct* OR "intra uterine" OR Intrafallopian) NEAR/3 inseminat*) OR (fertility NEAR/3 preservat*) OR ((embryo OR Zygote OR Gamete) NEAR/3 (disposit* OR Transfer* OR frozen)) OR ("in vitro" NEAR/3 fertilization*) OR ((intracytoplasmic OR "intra-cytoplasmic" OR single) NEAR/4 (sperm OR Gamete) NEAR/4 inject*) OR (ovulation NEAR/3 induc*) OR "In Vitro Oocyte Maturation*" OR (Oocyte NEAR/3 (Donation OR Retrieval)) OR Superovulation* OR (Assisted NEAR/3 reproduction*) OR (ovarian NEAR/3 (stimulation* OR hyperstimulation* OR reserv*)) OR "pregnancy rate" OR "implantation rate" OR "live birth rate" OR "pregnancy outcome"):ab,ti,kw
2. (((hCG OR "Human Chorionic Gonadotrophin" OR "oocyte collection*" OR "oocyte maturation*" OR "oocyte retrieval") AND (timing OR trigger*)) OR "hCG administration*" OR ((Oocyte OR ovulation) NEAR/4 trigger*) OR (stimulation NEAR/3 duration*)):ab,ti,kw
3. #1 and #2

**Cochrane Library**

#1 MeSH descriptor: [Reproductive Techniques, Assisted] explode all trees 3517

#2 (ART OR IVF OR ICSI OR COH OR IUI OR hyperovulation* OR ((artificial OR intrauterine* OR oviduct* OR "intra uterine" OR Intrafallopian) NEAR/3 inseminat*) OR (fertility NEAR/3 preservat*) OR ((embryo OR Zygote OR Gamete) NEAR/3 (disposit* OR Transfer* OR frozen)) OR ("in vitro" NEAR/3 fertilization*) OR ((intracytoplasmic OR "intra-cytoplasmic" OR single) NEAR/4 (sperm OR Gamete) NEAR/4 inject*) OR (ovulation NEAR/3 induc*) OR "In Vitro Oocyte Maturation*" OR (Oocyte NEAR/3 (Donation OR Retrieval)) OR Superovulation* OR (Assisted NEAR/3 reproduction*) OR (ovarian NEAR/3 (stimulation* OR hyperstimulation* OR reserv*)) OR "pregnancy rate" OR "implantation rate" OR "live birth rate" OR "pregnancy outcome"):ti,ab,kw 28068

#3 #1 OR #2 28082

#4 (((hCG OR "Human Chorionic Gonadotrophin" OR "oocyte collection*" OR "oocyte maturation*" OR "oocyte retrieval") AND (timing OR trigger*)) OR "hCG administration*" OR ((Oocyte OR ovulation) NEAR/4 trigger*) OR (stimulation NEAR/3 duration*)):ti,ab,kw 2288

#5 #3 AND #4

**Web of Science**

1. TS=(ART OR IVF OR ICSI OR COH OR IUI OR hyperovulation* OR ((artificial OR intrauterine* OR oviduct* OR "intra uterine" OR Intrafallopian) NEAR/3 inseminat*) OR (fertility NEAR/3 preservat*) OR ((embryo OR Zygote OR Gamete) NEAR/3 (disposit* OR Transfer* OR frozen)) OR ("in vitro" NEAR/3 fertilization*) OR ((intracytoplasmic OR "intra-cytoplasmic" OR single) NEAR/4 (sperm OR Gamete) NEAR/4 inject*) OR (ovulation NEAR/3 induc*) OR "In Vitro Oocyte Maturation*" OR (Oocyte NEAR/3 (Donation OR Retrieval)) OR Superovulation* OR (Assisted NEAR/3 reproduction*) OR (ovarian NEAR/3 (stimulation* OR hyperstimulation* OR reserv*)) OR "pregnancy rate" OR "implantation rate" OR "live birth rate" OR "pregnancy outcome")
2. TS=(((hCG OR "Human Chorionic Gonadotrophin" OR "oocyte collection*" OR "oocyte maturation*" OR "oocyte retrieval") AND (timing OR trigger*)) OR "hCG administration*" OR ((Oocyte OR ovulation) NEAR/4 trigger*) OR (stimulation NEAR/3 duration*))
3. #1 and #2

**CNKI**

(SU%=辅助生殖+生殖治疗+胚胎移植+冻胚+冷冻胚胎+冻融胚胎+试管婴儿+促排+促排卵+人工受孕+鲜胚移植+体外受精+体外授精+人工授精+人工受精+胚胎植入+胞浆内单精子显微注射+胞浆内单精子注射+IVF+ICSI+卵巢反应不良+卵巢储备不良+植入率+妊娠率+着床率+活产率+活产结局+妊娠结局+卵巢低储备+诱导排卵+卵巢刺激 OR TKA=辅助生殖+生殖治疗+胚胎移植+冻胚+冷冻胚胎+冻融胚胎+试管婴儿+促排+促排卵+人工受孕+鲜胚移植+体外受精+体外授精+人工授精+人工受精+胚胎植入+胞浆内单精子显微注射+胞浆内单精子注射+IVF+ICSI+卵巢反应不良+卵巢储备不良+植入率+妊娠率+着床率+活产率+活产结局+妊娠结局+卵巢低储备+诱导排卵+卵巢刺激) AND (SU%=扳机+触发+hCG时机+卵母细胞成熟时机 OR TKA=扳机+触发+hCG时机+卵母细胞成熟时机)

**Wanfang**

主题:("辅助生殖" OR "生殖治疗" OR "胚胎移植" OR "冻胚" OR "冷冻胚胎" OR "冻融胚胎" OR "试管婴儿" OR "促排" OR "促排卵" OR "人工受孕" OR "鲜胚移植" OR "体外受精" OR "体外授精" OR "人工授精" OR "人工受精" OR "胚胎植入" OR "胞浆内单精子显微注射" OR "胞浆内单精子注射" OR "IVF" OR "ICSI" OR "卵巢反应不良" OR "卵巢储备不良" OR "植入率" OR "妊娠率" OR "着床率" OR "活产率" OR "活产结局" OR "妊娠结局" OR "卵巢低储备" OR "诱导排卵" OR "卵巢刺激") and 主题:("扳机" OR "触发" OR "hCG时机" OR "卵母细胞成熟时机")

**VIP**

(M=辅助生殖+生殖治疗+胚胎移植+冻胚+冷冻胚胎+冻融胚胎+试管婴儿+促排+促排卵+人工受孕+鲜胚移植+体外受精+体外授精+人工授精+人工受精+胚胎植入+胞浆内单精子显微注射+胞浆内单精子注射+IVF+ICSI+卵巢反应不良+卵巢储备不良+植入率+妊娠率+着床率+活产率+活产结局+妊娠结局+卵巢低储备+诱导排卵+卵巢刺激 OR R=辅助生殖+生殖治疗+胚胎移植+冻胚+冷冻胚胎+冻融胚胎+试管婴儿+促排+促排卵+人工受孕+鲜胚移植+体外受精+体外授精+人工授精+人工受精+胚胎植入+胞浆内单精子显微注射+胞浆内单精子注射+IVF+ICSI+卵巢反应不良+卵巢储备不良+植入率+妊娠率+着床率+活产率+活产结局+妊娠结局+卵巢低储备+诱导排卵+卵巢刺激) AND (M=扳机+触发+hCG时机+卵母细胞成熟时机 OR R=扳机+触发+hCG时机+卵母细胞成熟时机)

**CBM**

( "辅助生殖"[常用字段:智能] OR "生殖治疗"[常用字段:智能] OR "胚胎移植"[常用字段:智能] OR "冻胚"[常用字段:智能] OR "冷冻胚胎"[常用字段:智能] OR "冻融胚胎"[常用字段:智能] OR "试管婴儿"[常用字段:智能] OR "促排"[常用字段:智能] OR "促排卵"[常用字段:智能] OR "人工受孕"[常用字段:智能] OR "鲜胚移植"[常用字段:智能] OR "体外受精"[常用字段:智能] OR "体外授精"[常用字段:智能] OR "人工授精"[常用字段:智能] OR "人工受精"[常用字段:智能] OR "胚胎植入"[常用字段:智能] OR "胞浆内单精子显微注射"[常用字段:智能] OR "胞浆内单精子注射"[常用字段:智能] OR "IVF"[常用字段:智能] OR "ICSI"[常用字段:智能] OR "卵巢反应不良"[常用字段:智能] OR "卵巢储备不良"[常用字段:智能] OR "植入率"[常用字段:智能] OR "妊娠率"[常用字段:智能] OR "着床率"[常用字段:智能] OR "活产率"[常用字段:智能] OR "活产结局"[常用字段:智能] OR "妊娠结局"[常用字段:智能] OR "卵巢低储备"[常用字段:智能] OR "诱导排卵"[常用字段:智能] OR "卵巢刺激"[常用字段:智能]) and ( "扳机"[常用字段:智能] OR "触发"[常用字段:智能] OR "hCG时机"[常用字段:智能] OR "卵母细胞成熟时机"[常用字段:智能])
