# Supplementary material for: Meta-analysis of trigger timing in normal responders undergoing GnRH antagonist ovarian hyperstimulation protocol
Source: J Ovarian Res. 2024 Mar 5;17:56. doi: 10.1186/s13048-024-01379-3 (PMC10913352; doi:10.1186/s13048-024-01379-3)
Supplement: Supplementary file 2 — Supplementary Material 2 [file 13048_2024_1379_MOESM2_ESM.docx]

**Additioanl file 2.** Subgroups analysis of study design types for the comparison of the standard and delay trigger groups.

| **Outcome indicators** | **Forest plot** |
| --- | --- |
| **Estradiol level** | **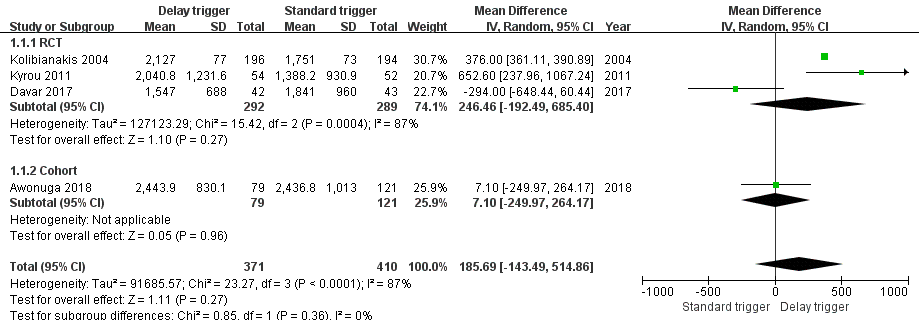** |
| **Progesterone level** | **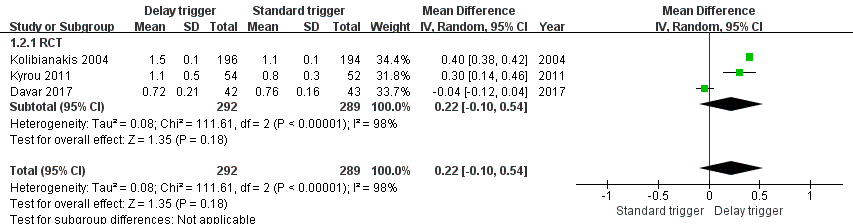** |
| **Gn duration** | 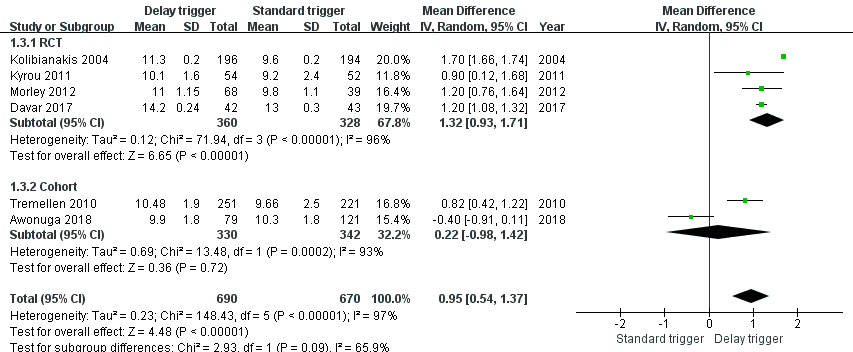 |
| **Total Gn dosage** | 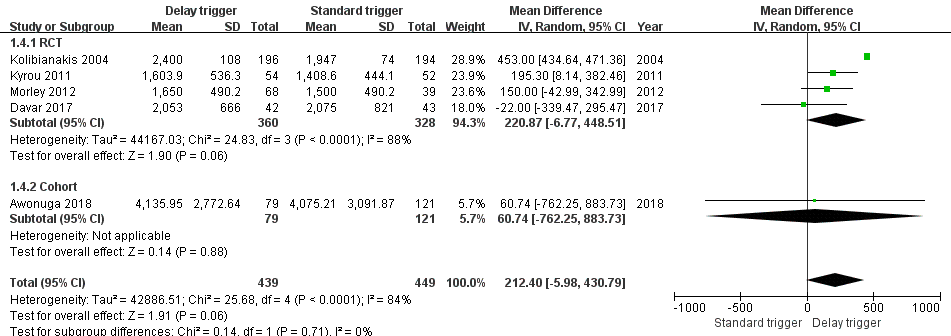 |
| **Oocytes retrieved** | 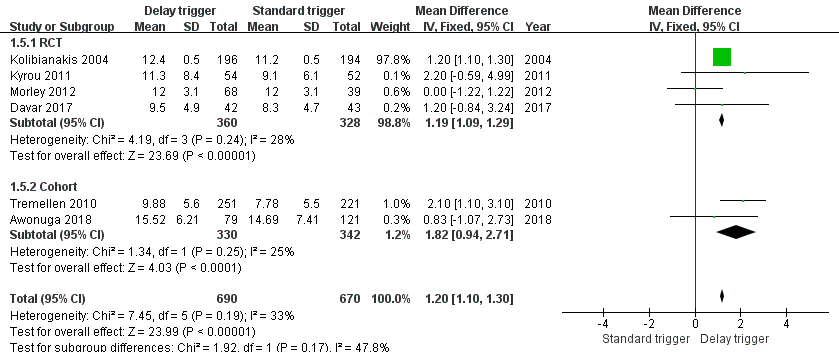 |
| **Fertilization rate** | 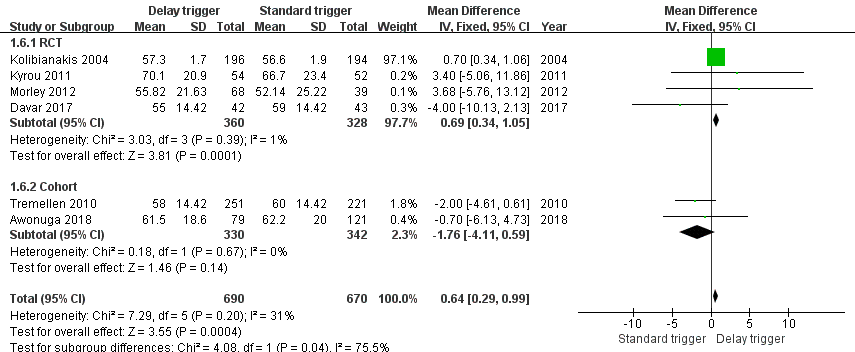 |
| **Number of embryos** | 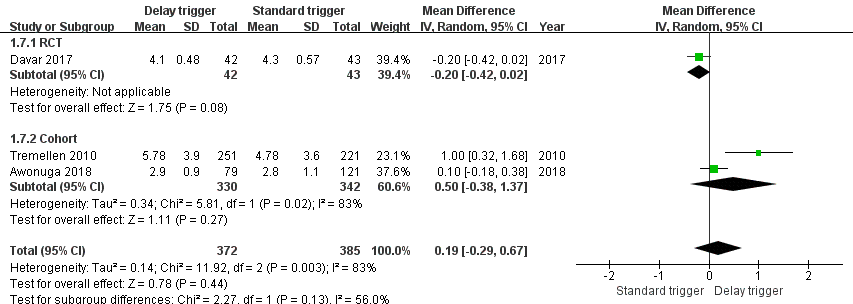 |
| **Clinical pregnancy rate** | 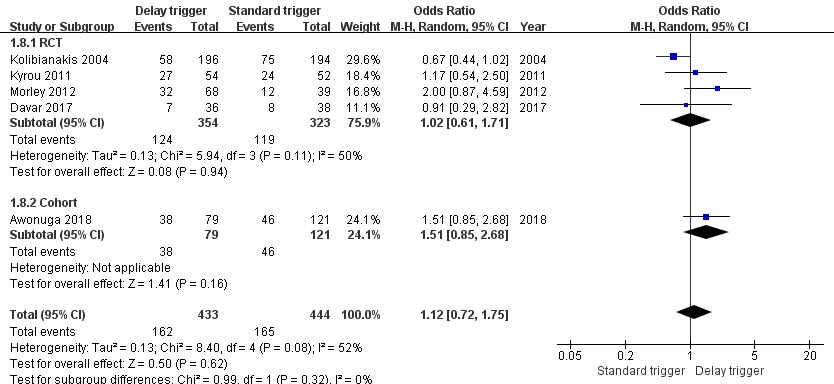 |
| **Live birth rate** | 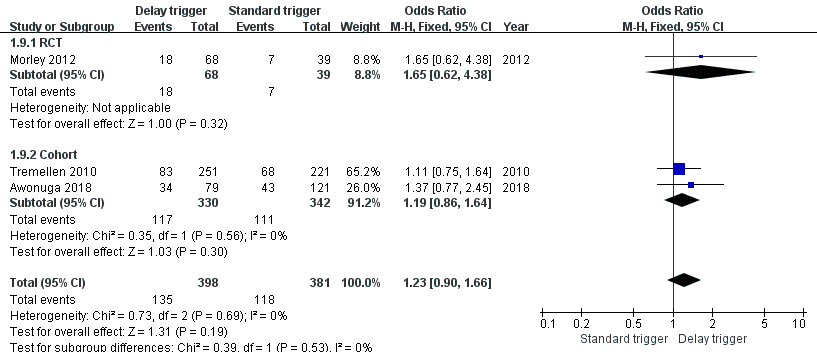 |
