# Supplementary material for: Meta-analysis of trigger timing in normal responders undergoing GnRH antagonist ovarian hyperstimulation protocol
Source: J Ovarian Res. 2024 Mar 5;17:56. doi: 10.1186/s13048-024-01379-3 (PMC10913352; doi:10.1186/s13048-024-01379-3)
Supplement: Supplementary file 3 — Supplementary Material 3 [file 13048_2024_1379_MOESM3_ESM.docx]

Additional file 3. Subgroups analysis of RCTs at low-risk of bias for the comparison of the standard and delay trigger groups.

| Outcome indicators | Forest plot |
| --- | --- |
| Estradiol level | 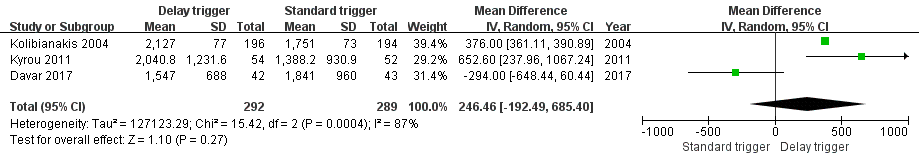 |
| Progesterone level | 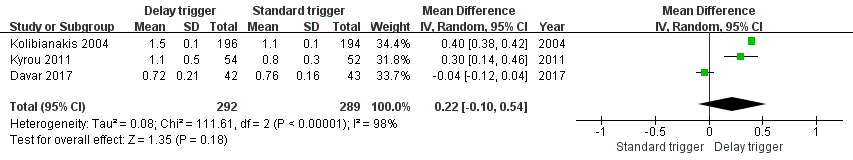 |
| Gn duration | 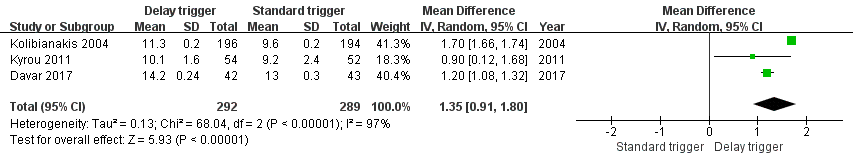 |
| Total Gn dosage | 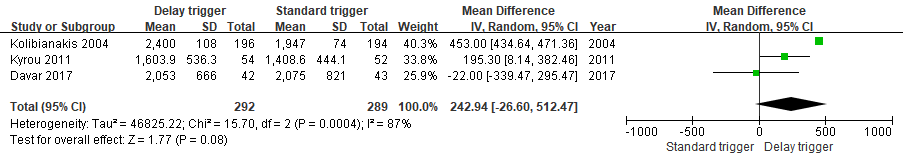 |
| Oocytes retrieved | 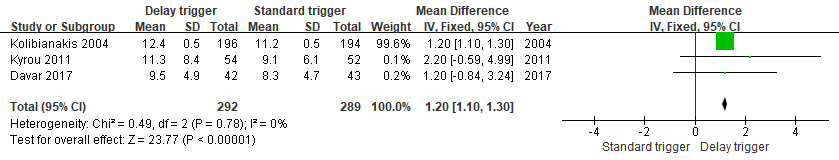 |
| Fertilization rate | 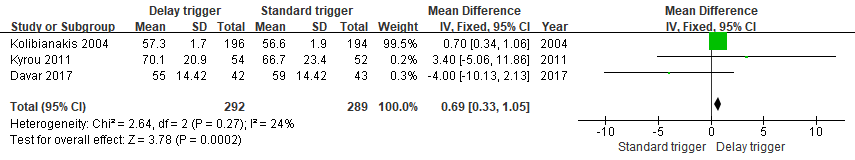 |
| Number of embryos | 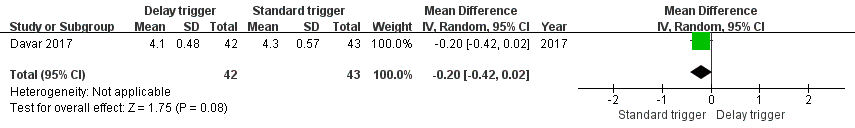 |
| Clinical pregnancy rate | 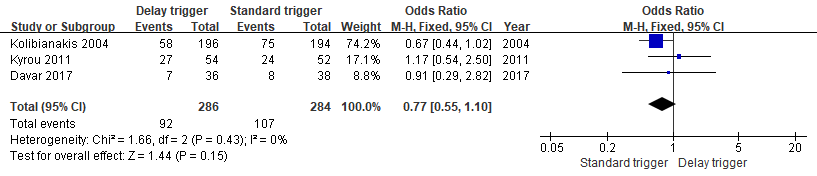 |
| Live birth rate | No RCT at low risk |
