# Supplementary material for: Meta-analysis of trigger timing in normal responders undergoing GnRH antagonist ovarian hyperstimulation protocol
Source: J Ovarian Res. 2024 Mar 5;17:56. doi: 10.1186/s13048-024-01379-3 (PMC10913352; doi:10.1186/s13048-024-01379-3)
Supplement: Supplementary file 4 — Supplementary Material 4 [file 13048_2024_1379_MOESM4_ESM.docx]

**Additional file 4.** Meta-analysis results for comparative studies of the standard and delay trigger groups.

| **Outcome indicators** |  | **Patients(cycle)** | **Statistical method** | **MD [95% CI]** | **P** |
| --- | --- | --- | --- | --- | --- |
| Estradiol level | Delay trigger VS Standard trigger | 371/410 | IV,Random | 185.69 [-143.49,514.86] | 0.27 |
|  | 24h-delay trigger VS Standard trigger | 96/95 | IV,Random | 172.93[-754.63,1100.50] | 0.71 |
|  | 48h-delay trigger VS Standard trigger | 196/194 | IV,Fixed | 376.00 [361.11,390.89] | <0.001 |
| Progesterone level | Delay trigger VS Standard trigger | 292/289 | IV,Random | 0.22 [-0.10,0.54] | 0.18 |
|  | 24h-delay trigger VS Standard trigger | 96/95 | IV,Random | 0.12 [-0.21,0.46] | 0.47 |
|  | 48h-delay trigger VS Standard trigger | 196/194 | IV,Fixed | 0.40 [0.38,0.42] | <0.001 |
| Gn duration | Delay trigger VS Standard trigger | 690/670 | IV,Random | 0.95 [0.54, 1.37] | <0.001 |
|  | 24h-delay trigger VS Standard trigger | 384/355 | IV,Random | 0.97 [0.68,1.26] | <0.001 |
|  | 48h-delay trigger VS Standard trigger | 227/233 | IV,Fixed | 1.70 [1.66,1.74] | <0.001 |
| Total Gn dosage | Delay trigger VS Standard trigger | 439/449 | IV,Random | 212.40 [-5.98,430.79] | 0.06 |
|  | 24h-delay trigger VS Standard trigger | 133/134 | IV,Fixed | 143.00 [12.85,273.14] | 0.03 |
|  | 48h-delay trigger VS Standard trigger | 227/233 | IV,Random | 324.31 [30.77,617.86] | 0.03 |
